# Evaluating Healthcare workers’ Mental health after four waves of COVID-19

**DOI:** 10.1192/j.eurpsy.2023.1676

**Published:** 2023-07-19

**Authors:** I. Sellami, A. Haddar, A. Abbes, A. Feki, N. Kotti, H. Halweni, M. L. Masmoudi, K. Jmal Hammami, M. Hajjaji

**Affiliations:** 1 occupational medecine, Hedi Chaker Hospital, university of Sfax; 2 occupational medecine, University of Sfax; 3 Rheumatology, Hedi Chaker Hospital, university of Sfax; 4 University of medecine, University of Sfax, Sfax, Tunisia

## Abstract

**Introduction:**

Health workers faced many challenges during the Pandemic of COVID-19. Continuous work stress and workload may affect their physical and mental health.

**Objectives:**

The study aimed to evaluate mental health among healthcare workers after the four peaks of COVID-19.

**Methods:**

We conducted a cross-sectional study on personnel working in a COVID-19 unit after four waves. We carried out a self-administrated questionnaire that included sociodemographic and professional data. To assess the level of depression, anxiety and stress symptoms we used the depression anxiety and stress scale (DASS 21).

**Results:**

The study included 69 healthcare workers. Their mean age was 31.7 ± 6.32 years and 52.2 % of them were male. Thirty-two per cent were technicians, 29% were administrators and 21,7% were nurses. Sixty-eight per cent had either direct or indirect contact with positive patients. The vast majority of them were vaccinated against SARS COV2 and 72,5 % received more than 1 dose. Regarding DASS-21, we found that 10,1% presented mild to moderate stress, 23% had mild to moderate anxiety and 16% had mild to moderate depression symptoms. Depression was correlated with the male gender (p=0.03).

**Conclusions:**

Our study showed a regression in terms of stress levels, anxiety, and depression among healthcare workers after the fourth wave, announcing the amelioration of mental health in case the pandemic gets to its end. A tight follow-up remains needed.

**Disclosure of Interest:**

None Declared

